# Consequences of Drought Stress Encountered During Seedling Stage on Physiology and Yield of Cultivated Cotton

**DOI:** 10.3389/fpls.2022.906444

**Published:** 2022-06-30

**Authors:** Tanzeela Rehman, Bushra Tabassum, Samina Yousaf, Ghulam Sarwar, Uzma Qaisar

**Affiliations:** ^1^School of Biological Sciences, University of the Punjab, Lahore, Pakistan; ^2^Institute of Botany, University of the Punjab, Lahore, Pakistan; ^3^Department of Cotton Section, Ayub Agricultural Research Institute, Faisalabad, Pakistan

**Keywords:** *Gossypium hirsutum*, drought stress, stress susceptibility index, yield potential, antioxidants, fibre quality

## Abstract

Survival of living organisms depends on the availability of water resources required for agriculture. In the current scenario of limited water resources, it is our priority to maximise the yield potential of crops with a minimum supply of available water. In this study, we evaluated seven cultivated varieties of *Gossypium hirsutum* (FH-114, FH-152, FH-326, FH-492, FH-942, VH-327 and FH-NOOR) for their tolerance, yield potential and fibre quality under water shortages. We also studied the effect of drought stress on osmoregulation, chlorophyll content, antioxidant (peroxidase and catalase) activity, lipid peroxidation and secondary metabolite accumulation in the varieties under study. It was revealed that three varieties (FH-114, FH-152 and VH-327) exhibited a lower stress susceptibility index and more tolerance to drought stress. All the varieties demonstrated enhanced proline and malondialdehyde content, but no significant change in chlorophyll content was observed under limited water supply. Antioxidant activity offered by catalase and phenolic content was enhanced in FH-492 whilst peroxidase activity increased in FH-114 and FH-326. Phenolic content was highest in FH-942 and decreased significantly in the remaining varieties. Ginning outturn of the cotton varieties increased in VH-327 (19.8%) and FH-326 (3.7%), was not affected in FH-114 and FH-492 and was reduced in FH-152, FH-942 and FH-NOOR. All cotton varieties tested showed an increase in micronaire thickness when exposed to drought stress as early as the seedling stage. This study highlights the evaluation and screening of cotton varieties for their response to drought stress in terms of yield and fibre quality when exposed to water shortages during plant development and can help in devising irrigation plans.

## Introduction

Water scarcity is the biggest emerging abiotic stress affecting all living organisms, especially plant growth and crop yield. On average 50% reduction in major crop harvest worldwide has been observed due to drought stress (Lamaoui et al., 2018). Cotton is a major fibre and oil producing crop cultivated in the temperate regions of the world. Pakistan is the fourth largest cotton producer and a 35% reduction in cotton production was observed in 2021 due to drought stress. Plant breeders and biotechnologists have to develop cotton genotypes whose yield is not adversely affected if exposed to water-limited conditions (Qaisar, 2012).

Irrigation water deficiency affects the morphological and physiochemical processes that result in hampered plant growth, development and yield (Fahad et al., 2017; Bozorov et al., 2018). Leaf functions are highly adversely affected under low water availability by producing harmful products such as reactive oxygen species (ROS). This is due to the imbalance between light capture and its utilisation by plant systems, following which production of superoxide anion, hydroxyl radicals, singlet oxygen and H_2_O_2_ occurs (Munné-Bosch and Penuelas, 2003). ROS attacks the cell machinery (Reddy et al., 2004), such as through the oxidation of photosynthetic pigments, and degradation of cell membrane lipids, proteins and nucleic acids (Reddy et al., 2004; Hasan et al., 2018). Malondialdehyde (MDA) content estimation can be used to assess membrane damage due to environmental stress and higher production of ROS (Singh et al., 2021). Plants can prevent drought stress and oxidative damage by producing antioxidant molecules (peroxidases, catalases, reductases and mutases) which scavenge ROS. Under drought stress, plants in addition to antioxidants also undergo the osmotic modification in cells by producing soluble solutes (Xiong and Zhu, 2002). The most common osmoregulatory solute in plants under drought stress is proline. It increases water flux in the plants by reducing the osmotic potential of the cell to maintain turgor and cell growth (Fumis and Pedras, 2002; Mafakheri et al., 2010; Nikolaeva et al., 2010).

Phenolics are secondary metabolites which accumulate under water deficit conditions (Hessini et al., 2022). This increase is attributed to the conversion of excess carbohydrates which are accumulated by the reduction of sugar transport to other plant parts. In order to maintain the balance between sugar source and sink, carbohydrates are converted to phenolics. Total phenolic content in plants is positively correlated with antioxidant activity. In the presence of large amounts of phenolics, antioxidant activity is significantly increased (Bettaieb et al., 2011; Gharibi et al., 2016). Moreover, free phenols are used to form covalent bonds with the carbohydrates of the cell wall preventing water loss from the cell (Hura et al., 2012).

Plants are known to undergo the above biochemical changes to cope with drought stress. However, diverse genotypes of cotton respond differently, determining the tolerance of a specific genotype. *G. hirsutum* contributes 90% of the world cotton production and is more resistant to harsh environments as compared to *Gossypium barbadense* (Hu et al., 2019). There is a need to identify *G. hirsutum* varieties which can perform better in terms of yield and quality of fibre when grown under limited water supply. The present study is designed to not only screen for drought sensitive and drought tolerant genotypes of *G. hirsutum* but also to study the dynamics of physiological processes when exposed to water shortages at the seedling stage. We evaluated seven cultivated varieties of cotton for their yield potential and fibre quality under limited water supply. We also studied stress susceptibility index, antioxidant activity, lipid peroxidation, osmoregulation and secondary metabolite accumulation to understand the responses which various genotypes exhibit when under drought stress. The outcomes of this study will help in devising irrigation strategies to maximise cotton yield using minimum water.

## Materials and Methods

### Plant Materials and Growth Conditions

Healthy seeds of seven cultivated varieties of cotton (FH-114, FH-152, FH-326, FH-492, FH-942, VH-327 and FH-NOOR) were collected from the Ayub Agricultural Research Institute (AARI), Faisalabad, Pakistan. Seeds were germinated on filter papers soaked with autoclaved distilled water. After 72 h, nearly 75% of seeds of all varieties germinated and were transferred to the soil mixture containing 50% silt, 32.5% sand and 17.5% clay in 20-inch diameter pots. Soil analysis was carried out for the determination of water holding capacity, electrical conductivity, pH, organic matter, and soil texture. Plant growth was carried out in the greenhouse at AARI, Faisalabad in October 2020. At least six biological replicates of each variety were maintained. Greenhouse temperature was maintained at 30 ± 5°C by using cooling and heating system. Metal halide illumination lamps (400 W) were used to maintain the day light intensity as previously described (Qaisar et al., 2010). Pots of each genotype were divided into two sets and the experiment was carried out using a randomised complete block design. All plants were watered regularly until the development of four true leaves. After 2 months post germination, seedlings of all varieties possessed at least four fully expanded true leaves. At this stage, water of one group of plants was withheld whilst the other group received anormal supply of water (half litre of water per pot on alternate days). After 38 days of water stress, wilting of leaves was visible in all varieties. After collecting morphological data and samples, all the plants were watered.

### Stress Susceptibility Index

At the onset of wilting of the leaves (38 days of stress) shoot length (SL) of control and water stressed plants of each genotype in both treatments was measured in centimetres (cm) by using the ruler from the ground level of plant to the highest fully expand leaf. The difference in the mean SL of stressed and non-stressed plants was used to assess the susceptibility of genotypes as previously described (Zahid et al., 2021). The measure of resistance based on phenotypic response under any stress in comparison with response under the optimal environment is termed the SSI. It is used to illustrate the relative tolerance level for any stress by using the formula of Fischer and Maurer (1978).

### Analysis of Physiological Metabolites

Plant leaves were harvested at the wilting stage and examined for various biochemical attributes including proline content, chlorophyll content and total phenolics. Proline content was measured according to the protocol of Bates et al. (1973) with a slight modifications. 0.5 g of fresh leaf powder was homogenised in 3% sulfo-salicylic acid and was centrifuged at 7,000 rpm for 10 min. The supernatant was mixed with the same volume of glacial acetic acid and acid ninhydrin reagent. Then, it was heated at 100°C for 1 h. After cooling, 4 ml of toluene was added and mixed well. The upper layer was collected, and absorbance was measured at 520 nm. Total chlorophyll content was measured by using Arnon’s protocol (Arnon, 1949). 0.5 g fresh plant tissue was ground and mixed with 80% acetone. After centrifugation at 10,000 rpm for 15 min, the supernatant was separated to record its absorbance at 645 and 663 nm by spectrophotometry (SP-300, OPTIMA. Inc). Total phenolics were evaluated by homogenising 0.5 g fresh leaf tissue in 80% acetone. Centrifugation was performed at 12,000 rpm for 15 min and supernatant (0.1 ml) was mixed with 2 ml water and 1 ml Folin–Ciocalteu’s phenol reagent. Later, 5 ml of 20% Na_2_CO_3_ was added. Absorbance was calculated at 750 nm and total phenolics were measured by comparing with tannic acid standards (Julkunen-Tiitto, 1985; Rafiq et al., 2020).

### Analysis of Antioxidants

Catalase activity, malondialdehyde content and peroxidase enzyme activity of leaf samples of both irrigated and drought treated samples was estimated. Catalase activity was measured according to Abe et al. (2003) with few modifications. 0.5 g Fresh leaf powder was mixed with 50 mM potassium phosphate buffer (pH 7.0) and centrifuged at 12,000 rpm for 20 min. 100 μl of the supernatant was mixed with 0.75 M H_2_O_2_. Change in absorbance at 240 nm was measured with a 20 s interval for 2 min. Malondialdehyde content was estimated by thiobarbituric acid (TBA) reaction (Heath and Packer, 1968). 0.5 g Fresh leaf powder was mixed with 10 ml of 0.1% TCA and centrifuged. The supernatant was mixed with 20% TCA and 0.5% TBA. The solution was incubated at 100°C for half an hour and placed on ice to stop the reaction. The absorbance was measured at 532 nm. The effect of non-specific turbidity was removed by deducting absorbance at 600 nm. The extinction coefficient of 155 mM^−1^ cm^−1^ was used for MDA content determination. Peroxidase activity was determined according to Chance and Maehly (1955). Plant leaves were ground in 50 mM potassium phosphate buffer (pH 7.0), centrifugation was carried out at 12,000 rpm for 20 min. The reaction mixture (3 ml) contained 50 mM phosphate buffer (pH 7.0), 0.1 ml H_2_O_2_ (40 mM), 0.1 ml guaiacol (20 mM) and 0.1 ml of above supernatant. An increase in absorbance was measured spectrophotometrically at 470 nm, with 20 s interval for 2 min (Rafiq et al., 2020) using a spectrophotometer (SP-300, OPTIMA. Inc).

### Yield and Fibre Quality Measurements

At the onset of wilting, leaf samples were collected for biochemical analysis and all the plants (control and drought stressed) were watered. Good agricultural practises were applied until fibre maturation. Yield parameters like number of bolls per plant, average boll weight and ginning outturn (GOT) percentages were measured. Mature fibres were collected from the control plants and the plants exposed to drought stress. Fibre quality parameters (micronaire, upper half mean length, breaking strength, uniformity index, short fibre, fibre elongation and fibre maturity) of each variety were assessed using High Volume Instrument (USTER HVI 1000) at Ayub Agricultural Research Institute, Faisalabad using standard protocols (Qaisar et al., 2017).

### Statistical Analysis

The data was statistically analysed by paired *T*-test for the comparison of control and drought treated samples whilst analysis of variance (two-way ANOVA) was used for the completely randomised block design using Statistix 10 Analytical software (Statistix, FL, United States). Tukey HSD was used for multiple comparisons to find significant differences among genotypes and treatments (*p* < 0.05). Principal component analysis and agglomerative hierarchical clustering was performed by using XLSTAT software in Microsoft Excel. SSIs were calculated using the formula given by Fischer and Maurer (1978).

## Results

### Drought Susceptibility Index of Cultivated Genotypes of Cotton

**Figure 1:**
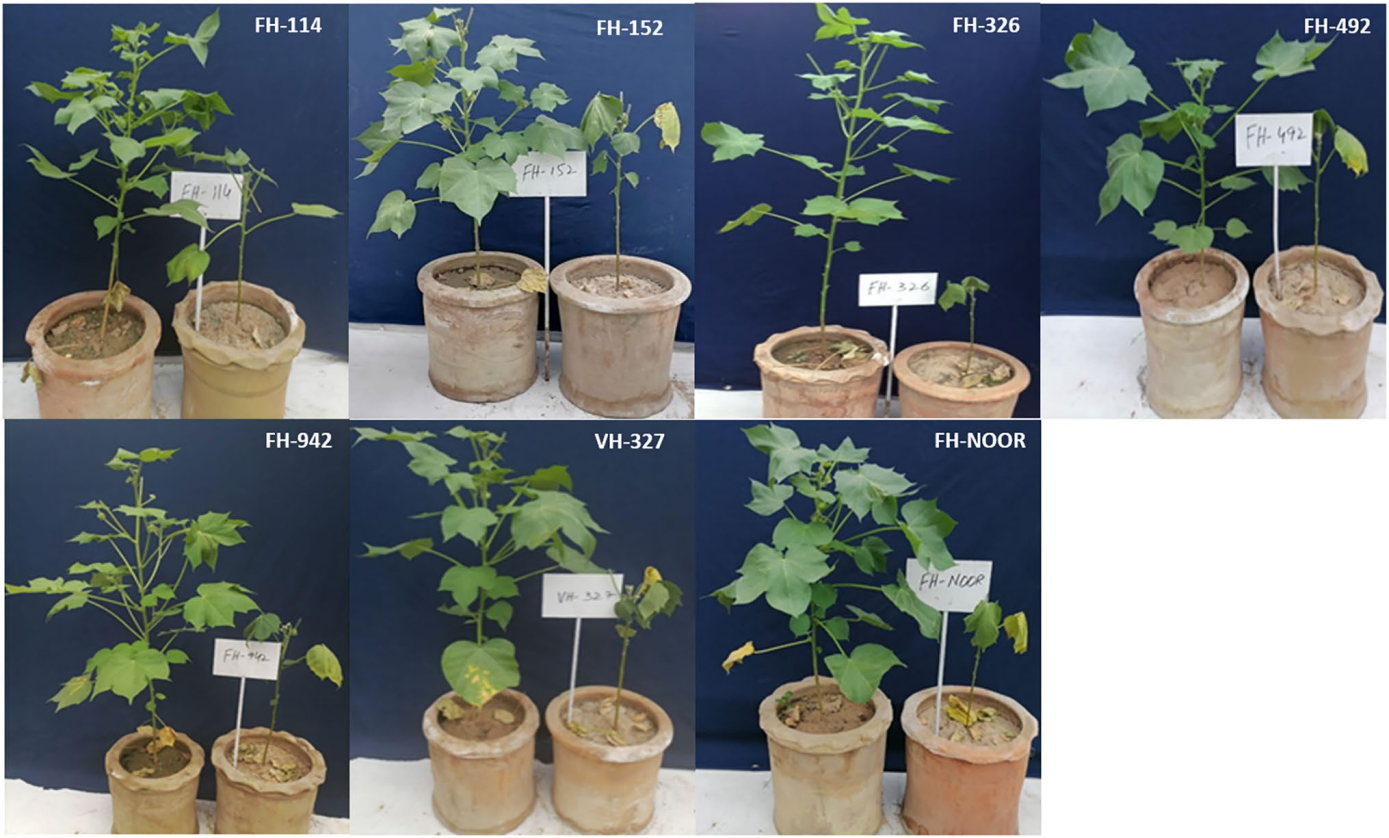
Effect of water scarcity on the phenomics of seven cultivated varieties of *Gossypium hirsutum*. The shown plant is the representative of all replicates.

**Figure 2:**
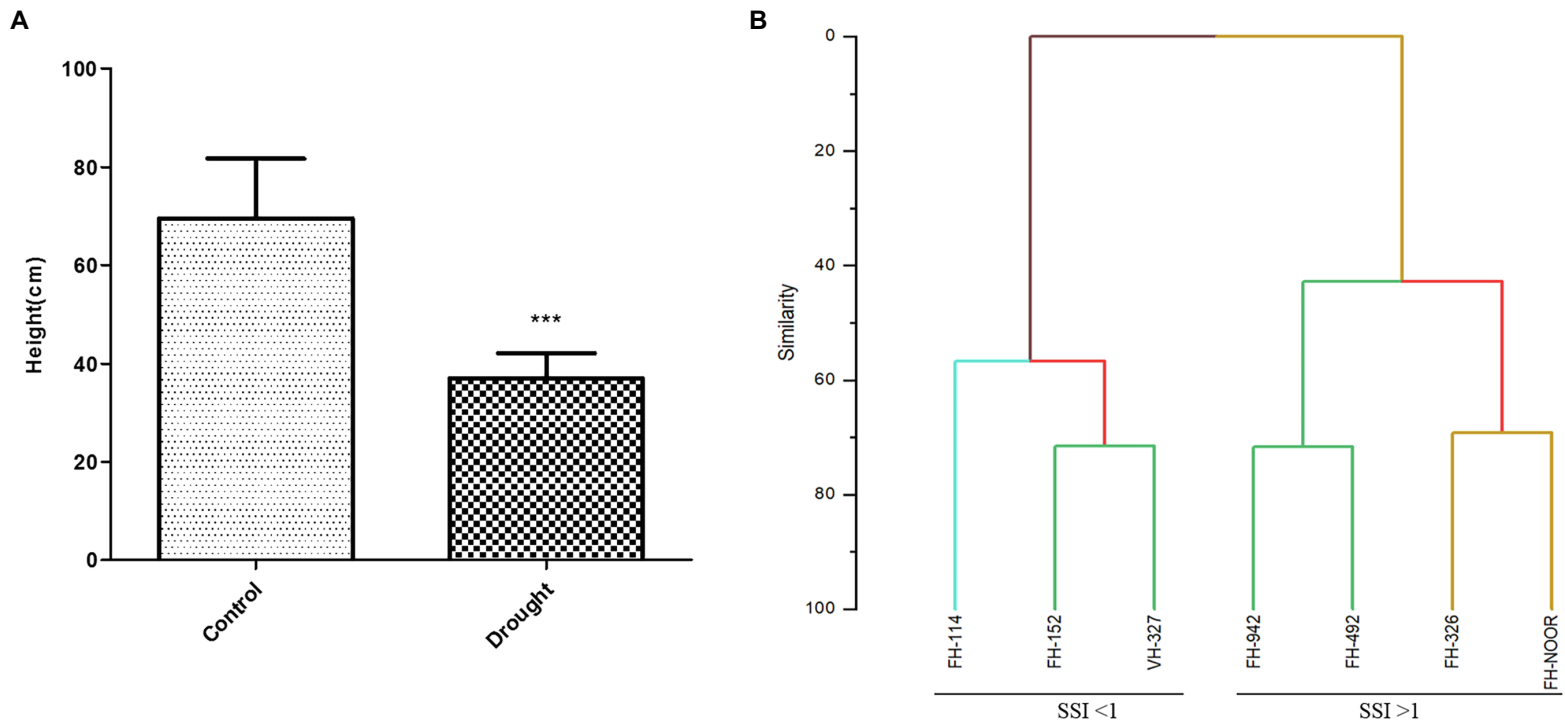
Effect of drought stress on the shoot length of cultivated varieties of *Gossypium hirsutum*. Bar heights represent the average of replicated values and error bars represent standard deviation **(A)**, sorting of cotton varieties on the basis of stress susceptibility index **(B)**.

**Figure 3:**
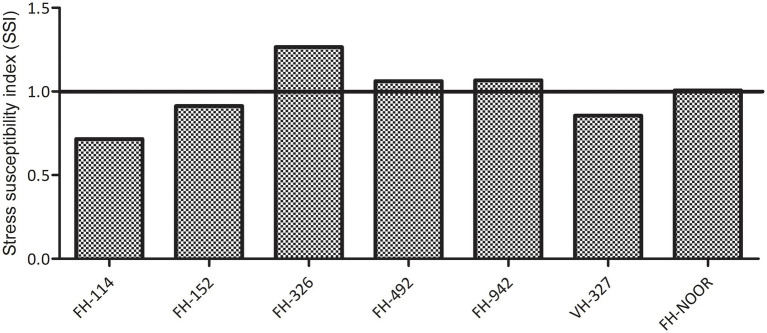
Stress susceptibility index of cultivated varieties of *Gossypium hirsutum* on exposure to drought.

Seven cultivated genotypes of cotton (FH-114, FH-152, FH-326, FH-492, FH-942, VH-327 and FH-NOOR) were validated for fitness to grow in the areas affected by episodes of drought stress. The group of plants for which irrigation was stopped until wilting at the seedling stage, exhibited stunted growth and reduction in shoot length (SL) as compared to regularly watered plants (Figures 1, 2A). Maximum reduction was observed in FH-326 (59.29%) and minimum in FH-114 (33.54%) as shown in Table 1. Stress susceptibility index (SSI) was analysed to identify the drought tolerant and drought sensitive genotypes of cotton according to the protocol of Fischer and Maurer (1978). SSI values for genotypes FH-114, FH-152 and VH-327 were <1, so they were classified as drought tolerant and for the FH-326, FH-492, FH-942 and FH-NOOR SSI was >1 and hence categorised as the drought sensitive varieties (Table 1; Figures 2, 3).

**Table 1:** Stress susceptibility indices for shoot length (SL) under water limited conditions in comparison with irrigated control.

Varieties	SL of irrigated plant (cm ± SD)	SL of drought stressed plant (cm ± SD)	Decrease in SL (%)	SI	SSI
FH-114	54.667 (±3.786)	36.33 (±0.577)	33.543	0.468	0.716
FH-152	75.667 (±4.509)	43.33 (±0.557)	42.73		0.913
FH-326	84.33 (±3.055)	34.33 (±3.512)	59.29		1.266
FH-492	62.33 (±1.155)	31.33 (±4.509)	49.73		1.062
FH-942	84.33 (±1.528)	42.22 (±4.041)	49.93		1.067
VH-327	67.33 (±1.528)	40.33 (±2.309)	40.145		0.856
FH-NOOR	58.00 (±1.00)	30.66 (±3.05)	47.137		1.007

SL, Shoot length; SD, Standard Deviation; SI, Stress index; SSI, Stress susceptibility index.

Agglomerative hierarchical clustering analysis was performed to categorise these genotypes in various groups. The hierarchical clustering classified genotypes in two groups based on SSI under drought stress (Figure 2B). Four genotypes (FH-326, FH-492, FH-942 and FH-NOOR) exhibiting higher SSI value were clustered together whilst three genotypes (FH-114, FH-152 and VH-327) separated in a cluster. Genotypes displaying higher SSI were subdivided in two groups. FH-NOOR and FH-326 were bundled in one subgroup whilst FH-492 and FH-942 in the second (Figure 2B). FH-114, exhibiting minimal stress impact at the seedling stage, proved to be the most tolerant genotype in terms of plant morphology.

### Physiological Responses of Cotton Varieties on Exposure to Drought Stress

**Figure 4:**
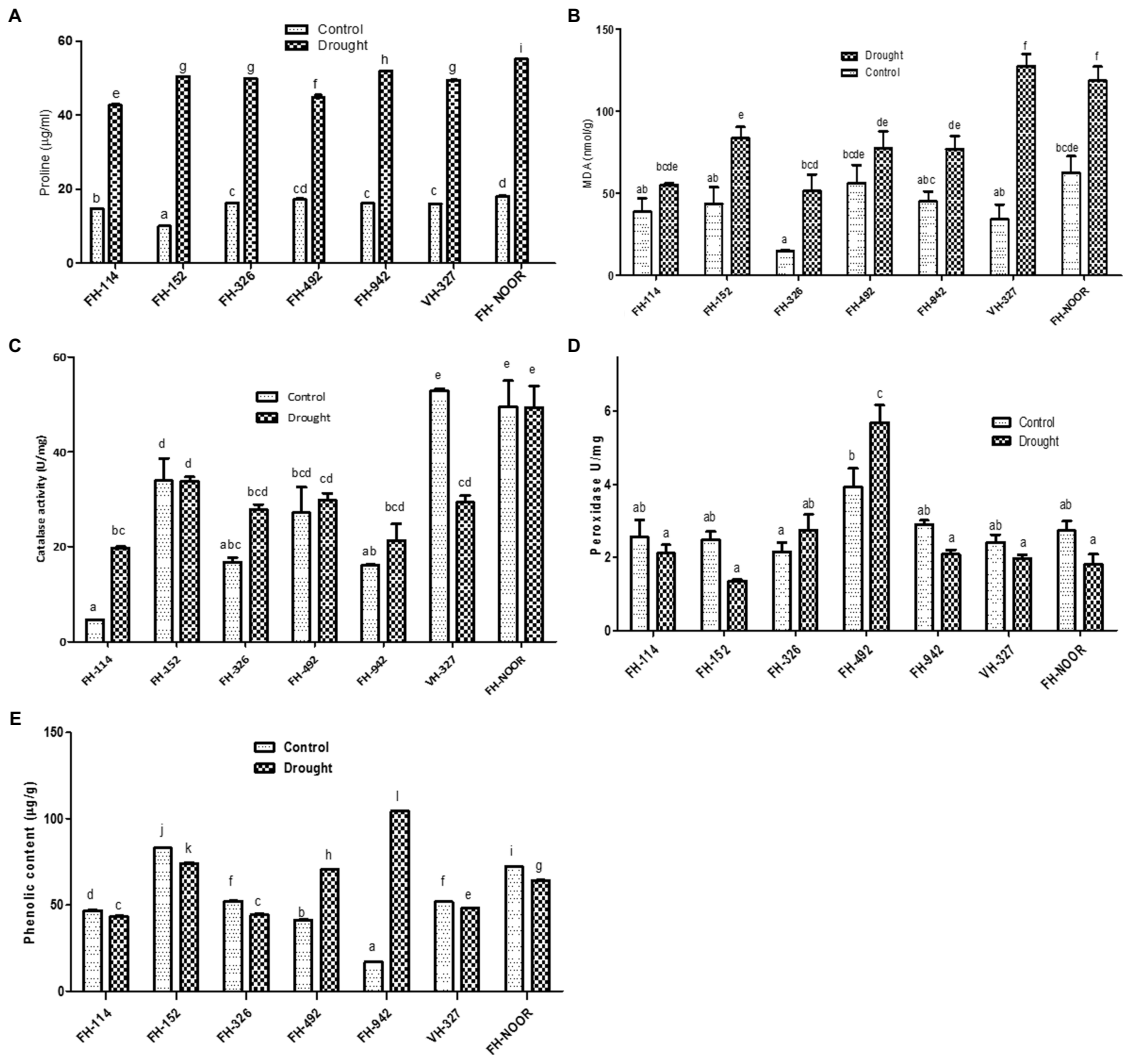
Effect of drought on metabolites of cultivated varieties of *Gossypium hirsutum*. Proline content **(A)**, malondialdehyde content, MDA **(B)**, catalase activity **(C)**, peroxidase activity **(D)** and total phenolic content **(E)**. Values were means ± SE of biological replicates (*n* = 3) while letters on bars indicate Tukey HSD comparison. Similar letters indicate no significant difference while non-similar letters indicate significant differences with 95% confidence level.

Proline content, which is an indicator of osmotic stress, was measured in drought treated and irrigated plants to investigate their physiological status. Proline content increased significantly in all varieties under water scarcity in comparison with the control (Figure 4A). Maximum increase (5 fold) was observed in FH-152. The highest level was observed in FH-NOOR both in control and stressed plants, i.e., 18.03 ± 0.2 and 55.15 ± 0.096, respectively. The lowest level (42.67 ± 0.4) was observed in FH-114 under drought stress conditions.

Malondialdehyde content (MDA) which is a lipid peroxidation marker and an indicator of oxidative damage to the cell membrane was investigated in various cultivated varieties of cotton under drought stress in comparison with control. All genotypes under study showed a significant increase in MDA content except FH-114 and FH-492, for which any increase was not statistically significant (Figure 4B). This decrease in the oxidative damage is in accordance with the reduced stress symptoms observed in the morphology of FH-114 plants under drought stress (Figure 1). Maximum increase (3.7 fold) was observed in VH-327 followed by FH-326 which exhibited 3.4 fold increase under stress conditions (Figure 4B). FH-NOOR showed maximum levels of MDA (62.56 nmol/g) under control conditions and this level was further increased under stress condition (118.5 nmol/g).

Total phenolic content significantly decreased in all genotypes under drought stress except in FH-492 and FH-942, where it increased 1.7 fold and 6.1 fold, respectively, (Figure 4E). Since phenolics are directly correlated with the antioxidant activity of plants, we studied the effect of drought stress on the peroxidase and catalase activities of drought treated and irrigated plants of all seven cultivated varieties under study. Catalase activity significantly increased in FH-114 and FH-326 whilst a significant decrease was observed in VH-327 (Figure 4C). Peroxidase activity changed under drought stress in all genotypes but the change was statistically non-significant except in FH-492, where activity increased (Figure 4D).

Total chlorophyll content did not differ significantly across all genotypes under water scarcity except in FH-492, in which total chlorophyll content slightly increased under drought stress (Supplementary Figure S1).

### Principal Component Analysis of Biochemical Response of Cotton to Drought Stress

**Figure 5:**
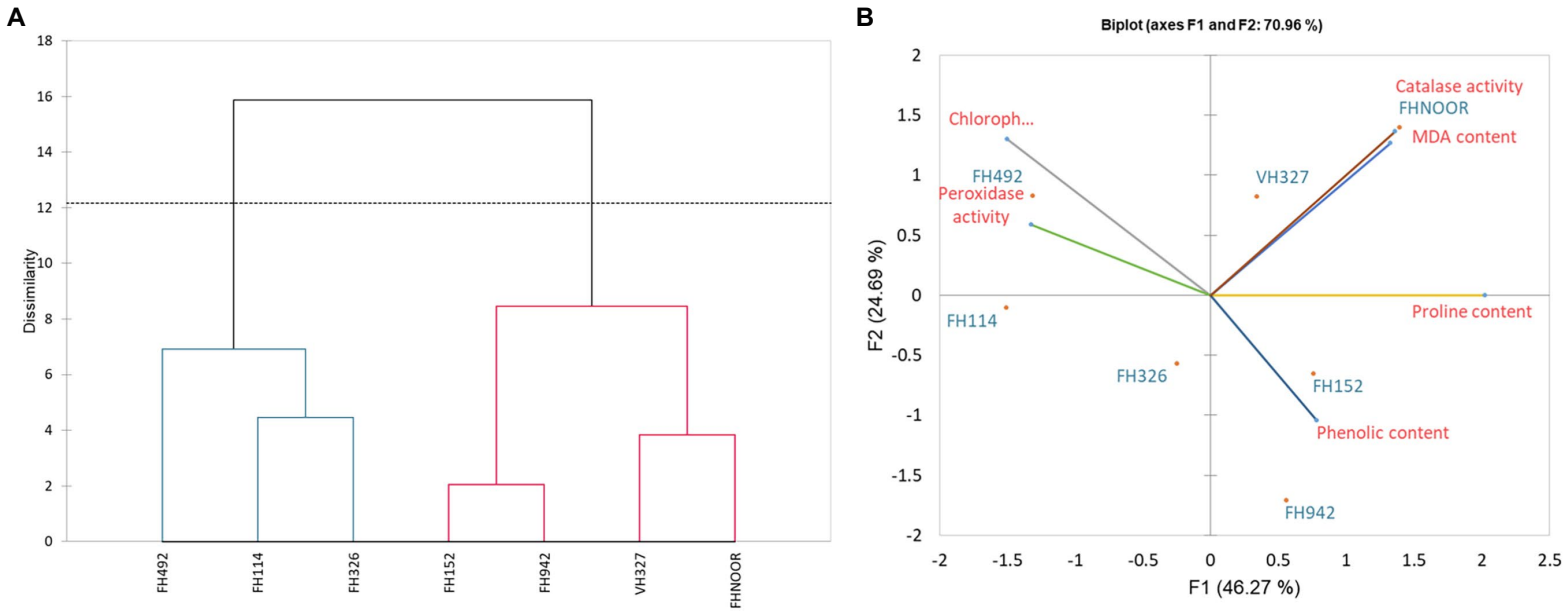
Dendrogram representing the clustering **(A)** and Biplot showing contribution **(B)** of various physiological parameters in drought response.

In order to evaluate and correlate the biochemical behaviour of cultivated cotton varieties exposed to drought stress, we performed Eigen analysis to identify the structure of our data. Three components representing an Eigen value more than one were identified as principal components (F1, F2 and F3) and they contributed 87.69% of the total variations (Figure 5). F1 contributed maximum variation (46.269%) followed by F2 (24.689%) and F3 (16.732%) under drought stress conditions. Phenolic content, proline content, chlorophyll content and peroxidase activity showed more contribution in F1 whilst F2 showed significant contribution with phenolic content, proline content and peroxidase activity. F3 demonstrated contribution with the phenolic content and MDA content (Table 2).

**Table 2:** Principal component analysis of biochemical modulations in cotton under drought stress.

	F1	F2	F3	F4	F5	F6
Eigenvalue	2.776	1.481	1.004	0.469	0.208	0.061
Variability (%)	46.269	24.689	16.732	7.822	3.474	1.013
Cumulative (%)	46.269	70.958	87.690	95.513	98.987	100.000

Since F1 and F2 were responsible for more than 70% of the variation among the genotypes under water-stressed conditions, a biplot between F1 and F2 was constructed. The biplot revealed that biochemical and genotypic data are superimposed under drought stress (Figure 5B). Distance between the biochemical parameters with respect to F1 and F2 are responsible for the variation among the genotypes under drought stress. It demonstrates that proline content, chlorophyll content, phenolic content, MDA content and peroxidase activity are contributors to adaptability of genotypes under drought stress (Table 3). Genotypes FH-114, FH-326 and FH-492 are correlated with each other on the basis of response to water scarcity in terms of antioxidant activity, osmolyte production, lipid peroxidation and chlorophyll content (Figure 5A). Genotypes VH-327, FH-NOOR, FH-492 and FH-942 are clustered in one group which is subdivided into two groups on the basis of similarity in physiological response to drought stress (Figure 5A).

**Table 3:** Contribution of biochemical variables (%) in principal components under drought conditions.

	F1	F2	F3	F4	F5
Chlorophyll	18.241	25.670	1.308	6.666	26.867
Proline content	33.261	0.000	0.917	6.354	1.000
MDA content	14.266	24.350	0.718	42.979	15.962
Peroxidase activity	14.170	5.195	41.445	12.244	26.905
Phenolic content	5.006	16.415	54.623	8.272	14.311
Catalase activity	15.056	28.370	0.990	23.486	14.955

### Effect of Drought Stress on Cotton Yield and Fibre Quality

The cotton plant produces cotton fibre and seeds which are enclosed in a boll. In order to evaluate the effect of water shortages during early plant development on the cotton yield, we investigated number of bolls per plant, average boll weight and ginning outturn (GOT) in commonly cultivated varieties of *G. hirsutum* (Table 4). The effect of drought stress on the yield was diverse among the different genotypes of cotton. Significant decrease in number of bolls per plant was observed in FH-114 (42.85%), FH-326 (72%) and FH-942 (67.85%) whilst in FH-NOOR a statistically non-significant decrease was observed. In FH-152, FH-492 and FH-327, statistically significant increases in the number of bolls 72.22, 64.70 and 23.08%, respectively, was evident (Table 4). There was no significant change in the weight of bolls except for genotype FH-492 which showed 26.6% increase in comparison with irrigated plants. GOT decreased in all genotypes exposed to drought during the seedling stage, whilst VH-327 exhibited an increase of 19.81% and FH-326 (3.7%) whilst FH-492 did not show any significant change in GOT.

**Table 4:** Consequences of drought stress on the yield parameters on cultivated varieties of cotton (*Gossypium hirsutum*).

Varieties	Bolls/Plant (no.)		Avg Boll weight (g)		GOT (%)	
	Irrigated	Stressed	Irrigated	Stressed	Irrigated	Stressed
FH-114	35.0 ± 1.50	20.00 ± 0.5	3.60 ± 0.18	3.30 ± 0.3	38.03 ± 0.45	37.60 ± 0.36
FH-152	18 ± 1.00	31.00 ± 0.5	4.87 ± 0.06	4.53 ± 0.25	44.91 ± 0.16	42.45 ± 0.22
FH-326	25.00 ± 0.66	7.00 ± 0.2	3.83 ± 0.21	4.07 ± 0.06	38.35 ± 0.74	39.78 ± 0.8
FH-492	13.00 ± 0.10	16.00 ± 0.8	3.00 ± 0.1	3.80 ± 0.1	43.32 ± 0.17	42.87 ± 0.64
FH-942	28.00 ± 0.20	9.00 ± 0.25	4.20 ± 0.1	4.07 ± 0.06	43.05 ± 0.37	41.50 ± 0.44
VH-327	17.00 ± 0.43	28.00 ± 0.89	3.90 ± 0.05	3.57 ± 0.21	35.57 ± 0.55	43.17 ± 0.47
FH-NOOR	20 ± 0.50	21.00 ± 0.25	3.73 ± 0.21	4.07 ± 0.06	44.67 ± 1	38.25 ± 0.51

Fibre quality of cultivated varieties of *G. hirsutum* was significantly affected by drought stress during the seedling stage. Micronaire is an important indicator of fibre quality and lower values represent higher quality and fineness. Micronaire was significantly increased by drought stress in all varieties of cotton in comparison with the irrigated control (Table 5). Due to increased thickness of the fibre, strength of the fibre was also increased in all *G. hirsutum* varieties under study. No statistically significant affect was evident on the percentage of fibre maturity. Percentage of short fibres was increased in the varieties FH-114, FH-152 and VH-327, but reduced in all other varieties under drought stress.

**Table 5:** Consequences of drought stress on the fibre quality parameters of cultivated varieties of cotton (*Gossypium hirsutum*).

Varieties	Micronaire (μg/in)		UHML (mm)		Breaking strength (g/tex)		Uniformity index (%)		Short fibre (%)		Fibre elongation (%)		Fibre maturity (%)	
	Irrigated	Stressed	Irrigated	Stressed	Irrigated	Stressed	Irrigated	Stressed	Irrigated	Stressed	Irrigated	Stressed	Irrigated	Stressed
FH-114	3.88 ± 0.1	4.34 ± 0.1	27.48 ± 0.45	30.40 ± 0.87	26.87 ± 0.81	31.80 ± 0.1	83.92 ± 0.52	84.92 ± 0.08	6.70 ± 0.1	7.43 ± 0.15	5.73 ± 0.15	4.13 ± 0.15	87.00 ± 0.50	87.03 ± 1.55
FH-152	3.68 ± 1	3.83 ± 0.05	26.85 ± 0.36	29.97 ± 0.16	28.83 ± 0.06	32.92 ± 0.03	84.84 ± 0.42	85.90 ± 0.05	5.80 ± 0.1	6.13 ± 0.15	3.88 ± 0.1	4.40 ± 0.36	86 ± 1	86.04 ± 0.06
FH-326	3.76 ± 0.2	4.15 ± 0.13	27.48 ± 0.19	29.08 ± 0.51	28.60 ± 0.3	29.55 ± 0.58	84.45 ± 0.74	83.67 ± 0.95	7.53 ± 0.15	7.05 ± 0.4	4.68 ± 0.25	6.60 ± 0.35	86.04 ± 0.06	85.97 ± 1.12
FH-492	3.81 ± 0.1	4.19 ± 0.19	28.38 ± 0.06	29.42 ± 0.09	26.60 ± 0.26	31.30 ± 0.95	84.55 ± 0.56	85.90 ± 0.53	7.20 ± 0.2	6.07 ± 0.21	3.83 ± 0.15	5.20 ± 0.1	87.03 ± 1.55	85.97 ± 1.46
FH-942	4.42 ± 0.2	4.50 ± 0.20	26.33 ± 0.14	27.97 ± 0.06	26.33 ± 0.21	31.87 ± 0.72	82.83 ± 0.06	84.37 ± 0.06	7.78 ± 0.11	6.10 ± 0.36	5.37 ± 0.21	7.23 ± 0.21	87.03 ± 1.5	86.03 ± 0.15
VH-327	3.96 ± 0.02	4.50 ± 0.2	29.08 ± 0.51	25.63 ± 0.47	30.27 ± 0.15	33.19 ± 0.78	84.55 ± 0.56	84.33 ± 0.15	7.20 ± 0.2	7.70 ± 0.1	3.83 ± 0.15	5.60 ± 0.44	87.03 ± 1.55	86.97 ± 0.55
FH-NOOR	3.67 ± 0.25	4.50 ± 0.20	28.57 ± 0.31	29.08 ± 0.51	30.73 ± 0.31	33.19 ± 0.78	83.57 ± 0.59	85.30 ± 0.87	7.57 ± 0.21	4.90 ± 0.1	6.20 ± 0.21	5.07 ± 0.15	85.00 ± 1	87.95 ± 0.07

## Discussion

In this study seven cultivated varieties of *G. hirsutum* (FH-114, FH-152, FH-326, FH-492, FH-942, VH-327 and FH-NOOR) were used to study the morphological, physiological and biochemical changes in plants on exposure to drought stress. Significant variations in morphological, physiological and yield parameters were observed across genotypes. A significant decrease in shoot length (33–59%) was observed in all varieties on exposure to drought stress (Figure 2A). Genotypes FH-114, FH-152 and VH-327 showed less susceptibility whilst FH-326, FH-492 and FH-942 were more susceptible to drought conditions (Figures 2B, 3).

Drought stress induces a storm of ROS in plants causing progressive oxidative damage leading to cell death (Mahmood et al., 2020). In response, plants activate enzymatic and non-enzymatic antioxidant defence mechanisms to scavenge ROS. Proline is a part of the non-enzymatic antioxidant defence system and is an osmolyte whose accumulation is the first response to drought stress (Hasanuzzaman et al., 2019; Hussain et al., 2019; Mahmood et al., 2020). In this study, all varieties of cotton accumulated proline on exposure to drought stress (Figure 4A) to scavenge singlet oxygen and hydroxyl ions to maintain membrane integrity and also to maintain the turgor pressure of the cell (Ozden et al., 2009; Hayat et al., 2012). Malondialdehyde is produced as a result of peroxidation of membrane lipids and is considered to be an indicator of oxidative damage (Zhang et al., 2014). Lipid peroxidation increased in all varieties under drought stress. However, the magnitude of increase corresponded to the tolerance level of genotypes. Drought sensitive genotypes showed a prominent increase but drought tolerant genotypes exhibited a mild increase (Figure 4B), indicating less membrane damage. A recent study on water stress has also reported that proline and MDA are increased in leaves of Chinese cotton varieties on exposure to drought stress (Jie et al., 2020). The enzymatic ROS scavenging elements, catalase and peroxidase, were also studied in all genotypes under drought stress. Genotypes showed variation in anti-oxidative response but the sensitive genotypes showed either a decrease or no significant change in the production of catalase and peroxidase enzymes. Tolerant genotypes showed either elevated levels of one antioxidant enzyme or the other under drought stress (Figures 4C,D).

The genotypes which showed higher tolerance to water shortage at the morphological level demonstrated varying responses in antioxidant activity, lipid peroxidation and secondary metabolite accumulation. These biochemical modulations in response to drought stress determined its effect on fibre yield and quality. FH-114 displayed lowest SSI, minimal oxidative damage and enhanced catalase activity which partially compensated for the effects of drought stress. The antioxidant enzyme catalase degrades ROS, thus minimising oxidative damage caused by drought stress (Ullah et al., 2017). Although the number of bolls per plant was reduced, no significant change in the GOT was evident (Table 4). Micronaire and short fibre percentage increased significantly whilst fibre elongation was reduced. FH-152, which showed higher tolerance (lower SSI) to drought, did not initiate an antioxidant response (phenolics, catalase and peroxidase activity), resulting in a higher number of bolls under drought stress but lower GOT. At the onset of drought stress VH-327 had the highest lipid peroxidation indicating of oxidative stress, a decreased activity of antioxidants (catalase and peroxidase) and decreased levels of protective secondary metabolites (Figure 4). However, these stress symptoms and oxidative damage recovered when normal watering was resumed and plants exposed to water shortages produced a higher number of bolls (23.08%) and GOT (19.81%) in comparison with the regularly irrigated plants (Table 4). It can be concluded that an as yet explored mechanism, rather than activity of antioxidants, is playing a role in drought stress tolerance in VH-327.

**Figure 6:**
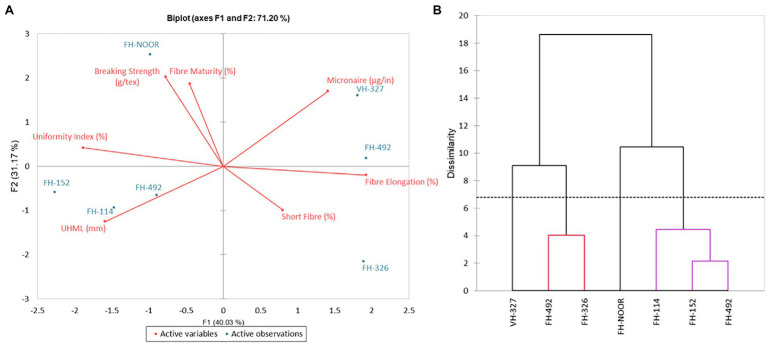
Principal component analysis **(A)** and dendrogram **(B)** of fibre quality parameters of cultivated varieties of *Gossypium hirsutum*.

FH-492 demonstrated a higher SSI (Figure 3) but a minimal increase in lipid peroxidation, possibly due to the increased levels of the H_2_O_2_ scavenging enzyme peroxidase. This variety compensated for the damage induced by drought stress on resumption of water availability and produced an increased number of bolls and average boll weight and no significant change in GOT (Table 4). In previous studies it has been observed that in drought tolerant genotypes of cotton, the level of peroxidase increased as compared to drought sensitive genotypes under water shortages (Zhang et al., 2014). Although FH-492 and FH-942 were grouped together on the basis of SSI and shoot length (Figures 1, 2B) they responded differently at the biochemical level and for yield parameters. FH-942 exhibited enhanced levels of proline and MDA content under drought stress but antioxidant response (catalase and peroxidase activity) was not triggered to scavenge the ROS (Figure 4) which resulted in a significant decrease in the number of bolls, average weight of bolls and GOT (Table 4). FH- Noor exhibited higher a SSI and lipid peroxidation due to a decrease in phenolics and antioxidant activity which resulted in decreased yield (Table 4). Thus, VH-327 and FH-492 showed tolerance to drought stress in terms of yield parameters; however, the response of both varieties towards different enzymatic and non-enzymatic activities varied. Micronaire is an important parameter to determine fibre quality of cotton, and drought stress during early plant development significantly thickened the micronaire of the fibre along with increasing fibre strength, whether an antioxidant response was initiated or not. Moreover, the percentage of short fibres increased on exposure to drought stress (Figure 6).

## Conclusion

In the current global scenario of water shortages, we have to introduce and promote to farmers only those varieties of cotton which can grow with a limited supply of water without compromising yield and fibre quality. Screening of all cultivated and non-cultivated genotypes should be done to minimise the amount of water used in the cultivation of major crops. In the current study, VH-327, FH-326 and FH-492 proved their potential for better yield and fibre quality under water shortages. These varieties should be promoted and used in the agriculture extension programmes for farmers. Similar studies should be done on the cultivated varieties of other crop plants to evaluate their yield potential under a limited supply of water for a more sustainable future agriculture.

## Data Availability Statement

The original contributions presented in the study are included in the article/Supplementary Material; further inquiries can be directed to the corresponding author.

## Author Contributions

TR and UQ planned the experiments, analysed data, and drafted the manuscript. TR and GS performed the main stream experiments. BT and SY analysed the data and revised the manuscript. All authors contributed to the article and approved the submitted version.

## Funding

This research was financially supported by the School of Biological Sciences, University of the Punjab, Lahore, Pakistan.

## Conflict of Interest

The authors declare that the research was conducted in the absence of any commercial or financial relationships that could be construed as a potential conflict of interest.

## Publisher’s Note

All claims expressed in this article are solely those of the authors and do not necessarily represent those of their affiliated organizations, or those of the publisher, the editors and the reviewers. Any product that may be evaluated in this article, or claim that may be made by its manufacturer, is not guaranteed or endorsed by the publisher.

## Supplementary Material

Supplementary Figure S1 Total chlorophyll content remained unaffected in drought stress. There was no significant increase or decrease in different genotypes of cotton under drought stress except in the FH-492 genotype. Values were means ± SE of biological replicates (*n* = 3; ^*^*p* < 0.05).
