# Supplementary figures and images for: Consequences of Drought Stress Encountered During Seedling Stage on Physiology and Yield of Cultivated Cotton
Source: Front Plant Sci. 2022 Jun 30;13:906444. doi: 10.3389/fpls.2022.906444 (PMC9280337; doi:10.3389/fpls.2022.906444)

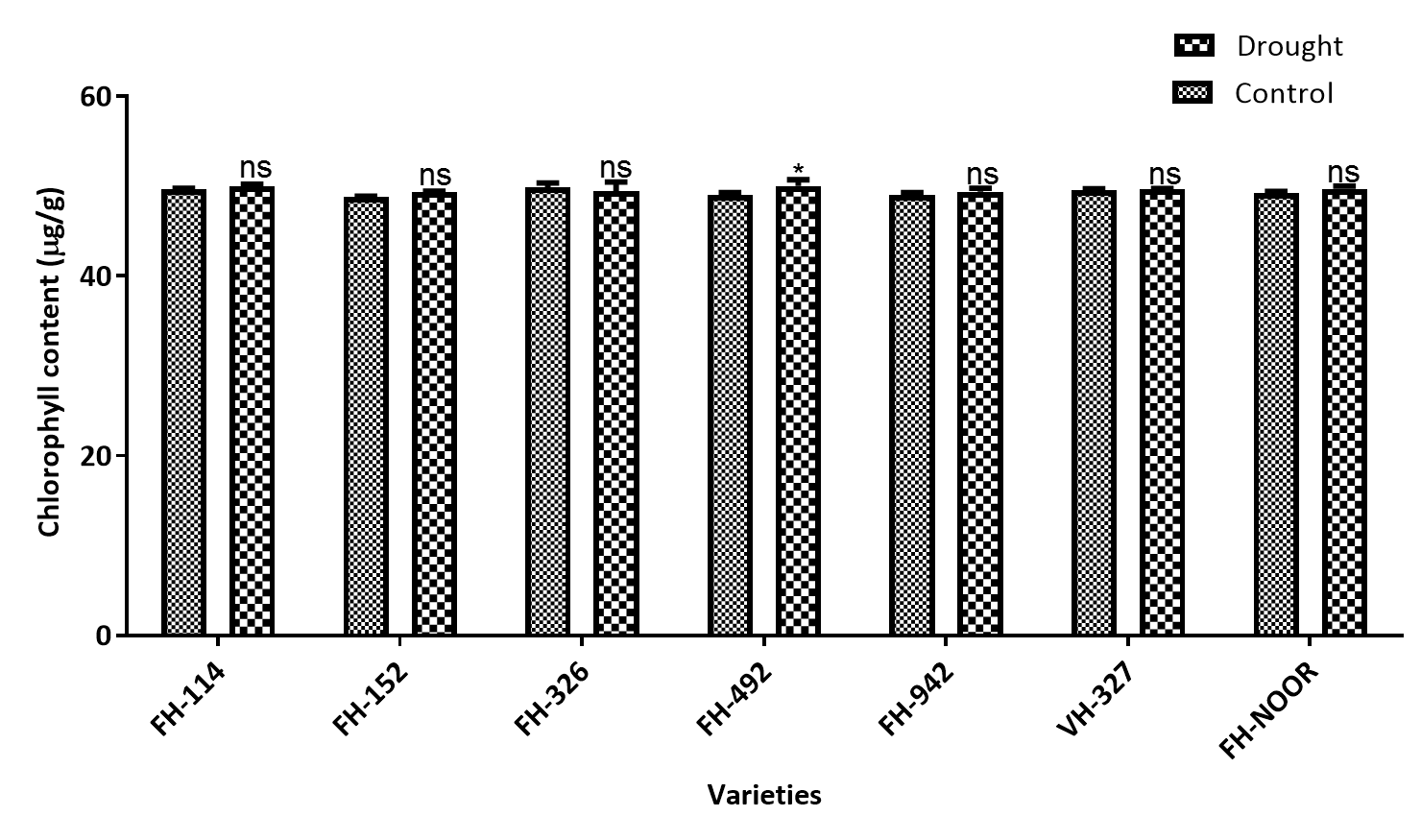

Supplement: Supplementary Figure S1 — Total chlorophyll content remained unaffected in drought stress. There was no significant increase or decrease in different genotypes of cotton under drought stress except in the FH-492 genotype. Values were means ± SE of biological replicates (n = 3; *p < 0.05). [file Image_1.TIF]
